# Clinical efficacy of various anti-hypertensive regimens in hypertensive women of Punjab; a longitudinal cohort study

**DOI:** 10.1186/s12905-020-01033-2

**Published:** 2020-08-01

**Authors:** Muhammad Umair, Mobasher Ahmad, Hamid Saeed, Zikria Saleem, Fatima Tauqeer

**Affiliations:** 1 Section of Pharmacology, University College of Pharmacy, University of the Punjab, Allama Iqbal Campus, Lahore, 54000 Pakistan; 2 Section of Pharmaceutics, University of the Punjab, Allama Iqbal Campus, Lahore, 54000 Pakistan; 3 University of Lahore, Lahore, Pakistan; 4 Institute of Health and Society, Department of Medicine, University of Oslo, Oslo, Norway

**Keywords:** Hypertension, Women, Punjab, Pakistan, Nifedipine-GITS, Losartan, Combination therapy

## Abstract

**Background:**

Gender wise differences exist in anti-hypertensive treatment outcomes, yet still un-explored in Pakistan. Thus, we aimed to estimate the clinical efficacy of four different anti-hypertensive regimens in hypertensive women of Punjab, Pakistan.

**Methods:**

A longitudinal cohort study of 12 months duration was conducted by enrolling 300 hypertensive women on four anti-hypertensive regimens. Chi-square for significance, logistic regression for association and multilevel regression for changes in outcomes were used.

**Results:**

Majority of subjects were < 60 years of age, weighing > 65 Kg, having family history, married and hailing from urban areas, with diabetes as the most common comorbidity. Hypertension, adjusted for covariates, was significantly associated with salt intake (OR:2.27, *p* <  0.01) and physical activity (OR;2.16, *p* <  0.01). High-risk subjects, compared to low-risk, were consuming more fat (OR;1.54), meat (OR; 2), salt (OR; 2.48) and even vegetables/fruits (OR;3.43). Compared to baseline, the maximum reduction in BP was observed with combination therapy, N-GITS+LTN + HCT (SBP; − 50.17, *p* <  0.01, DBP; − 16.55, *p* <  0.01), followed by N-GITS alone (SBP; − 28.89, *p* <  0.01, DBP; − 12.21, p <  0.01). Compared to baseline, adjusted for treatment effects, significant reductions in SBP (*low-risk****;*** − 17.92, *p* <  0.01 *high-risk*; − 19.48, *p* <  0.01) and DBP (*low-risk;* − 17.92, *p* <  0.01, *high-risk;* − 19.48, *p* <  0.01) were observed in low and high risk patients. Among all four cohorts, orthostatic hypotension and edema were common in N-GITS+LTN + HCT only, but variable effects were observed on biochemical values; urea, BSR and creatinine.

**Conclusion:**

In conclusion, compared to a single agent, combination therapy conferred improved BP controls followed by N-GITS alone in low and high risk women with manageable side effects.

## Background

Hypertension is a public health issue and a major cause of morbidity and mortality. It is responsible for almost 13% of all deaths and 3.7% total disability adjusted life years [1]. It is estimated that of all the deaths (17 million) globally due to cardiovascular diseases, 50% (~ 9.4 million) are due to complications related to hypertension [2]. It is now well documented that gender base differences exist in the pathophysiology of hypertension, probably due to age related differences in arterial tree between the sexes [3]. However, to date, the data is scarce that may demonstrate gender wise differences in blood pressure responses towards anti-hypertensive agents [4].

Literature regarding blood pressure control in both men and women are contradictory, a few studies suggest that women are more likely to be treated but less likely to achieve blood pressure control [5, 6]. However, age dependent relationship between blood pressure control and gender has been documented - poor blood pressure control in younger men and older women [7]. Numerous studies have shown that the risk of heart failure and mortality rate due to hypertension is greater in women compared to men [8, 9]. Contrary to standard guidelines, numerous observational studies have shown that both men and women are treated with different anti-hypertensive agents - women with diuretics or beta blockers and men with ACE inhibitors or calcium channel blockers [5, 6]. Women above 50 years of age exhibited greater protection from stroke on calcium channel blockers rather than using ACEIs [10]. According to European guidelines, calcium channel blockers are considered the only class of anti-hypertensive agents that can produce desirable effects in combination with other four classes of anti-hypertensive drugs [11].

Among others, Nifedipine gastrointestinal therapeutic system (N-GITS) provides sustained 24 h anti-hypertensive effect with no overt cardio-acceleration [12]. The efficacy of Nifedipine GITS has been established in numerous studies, alone or in combination, in hypertension and angina – supported by the outcomes of INSIGHT and ACTION trials [13, 14]. Results from a randomized control trial, ADVISE study, clearly demonstrated that blood pressure controls were better in Asian population, both males and females, on nifedipine GITS plus valsartan compared to higher doses of valsartan alone, even when stratified for smoking and systolic blood pressure (SBP) [15].

Despite higher prevalence of hypertension among women of Pakistan with associated risk of cardiovascular disease [16, 17], scanty of literature evidences exist regarding the clinical efficacy and safety of various anti-hypertensive agents in hypertensive women – not a single from Pakistan and almost negligible from South Asian region. Thus, the aim of the present study was to evaluate the clinical efficacy and safety of four anti-hypertensive regimens, namely; losartan (LTN), N-GITS (nifedipine-GITS), LTN + hydrochlorothiazide (LTN + HCT) and LTN + HCT + N-GITS, in low and high risk hypertensive women of Punjab, Pakistan.

## Methods

**Fig. 1:**
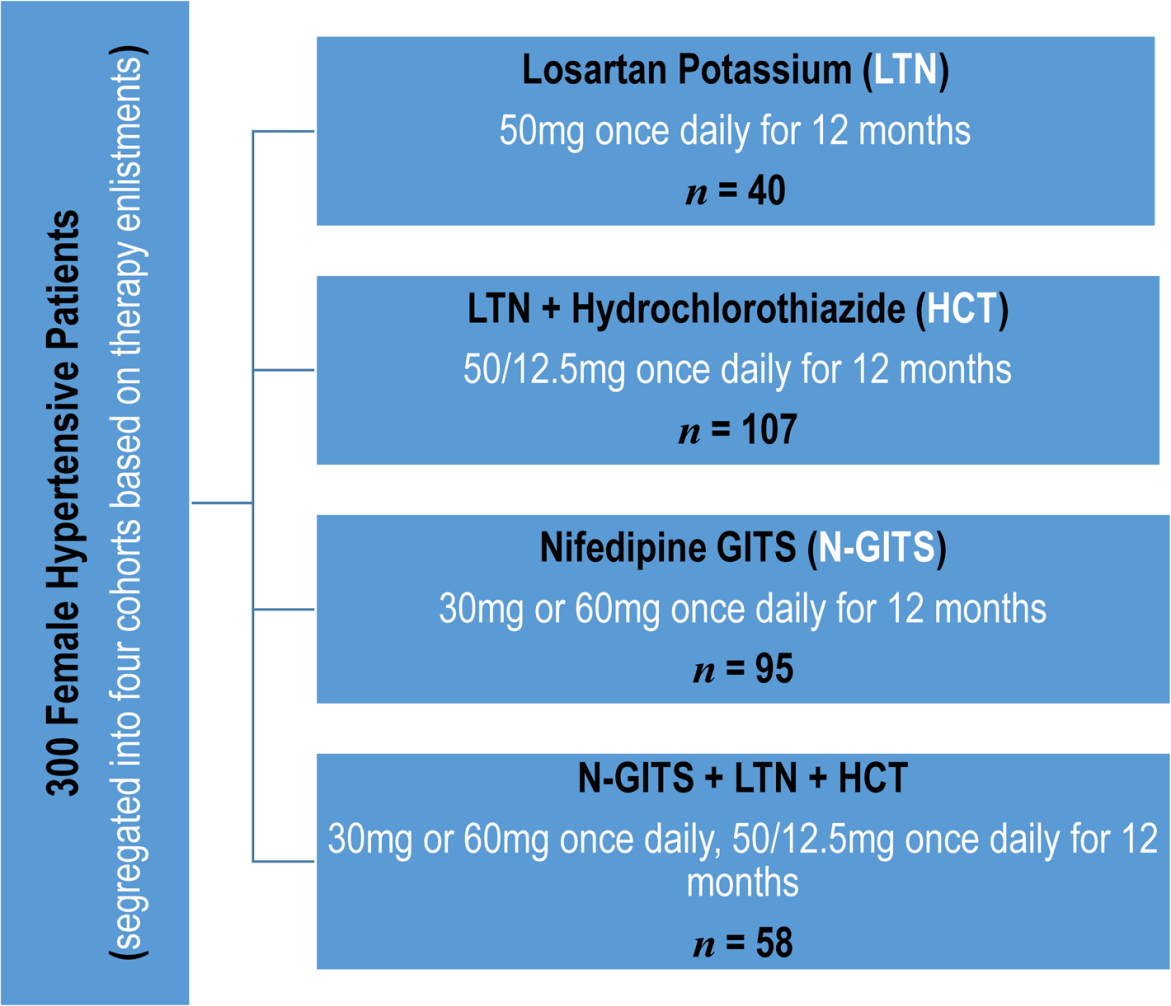
A Brief Layout of the Study Design

The layout of cohort study design is described in Fig. 1.

### Study design

A longitudinal cohort study was conducted by enrolling 300 hypertensive women from Fauji foundation hospital, Lahore. The study period was 1 year, i-e., June 2016–May 2017. Hypertension was documented as per clinician’s report – 150/90 mmHg or higher for patients 60 years or above without any comorbidity and 140/90 mmHg or higher for adults below 60 years of age as per JNC8 guidelines [18].

#### Sample size

Sample size was calculated based on disease prevalence (hypertension) [19] in Pakistani women, which was around 39%, as of June 2017 [20]. The sample size was found to be 363 using 95% confidence interval and 5% precision. However, we didn’t get more than 300 patients due specific therapy enlistment and selection of a single health facility (Fauji foundation hospital).

#### Study cohorts

As per study objectives, the study cohorts, i-e., Losartan (LTN) group (*n* = 40), Nifedipine-GITS (N-GITS) group (*n* = 95), LTN + hydrochlorothiazide (HCT) group (*n* = 107) and LTN + N-GITS + HCT group (*n* = 58), were identified from Hospital Information System (HIS) in consultation with the medical practitioner providing treatments to the patients reporting to the hospital for the year, 2016. Most of the patients in four cohorts were already on enlisted therapies before their enrollment in the study. After enrollments following study inclusion and exclusion criteria, the baseline clinical and laboratory parameters were recorded from patient’s medical files extracted via hospital information system.

#### Follow ups

After documenting baseline parameters, these 300 hypertensive women, started on four anti-hypertensive regimens mentioned above, were observed from June 2016 till May 2017 with total of 3 follow ups - each follow-up after every 3 months. All the parameters recorded at baseline were documented at each follow up to examine therapy effects.

#### Risk assessment

Subjects having co-morbid conditions were considered high risk, while hypertension alone cases were considered low risk.

### Study settings

The Fauji foundation hospital, established in 2001 and governed by Pakistani army, was selected because it’s one of the leading hospitals of Pakistan that receive new and referral hypertensive patients from all over the province, Punjab, with complete documentation of patient’s medical records [21]. It’s a 250 bedded hospital spread over an area of 6.5 acer in cantonment, Lahore, Punjab, Pakistan. Hospital provides free medical services and medication to its past and present employees – ex-military service men.

### Participants

A total of 300 subjects were registered in the study from Fauji Foundation Hospital, Lahore, Pakistan. The subjects were enrolled as per study inclusion and exclusion criteria.

#### Inclusion criteria

All hypertensive women above 18 years of age, with not more than two co-morbid conditions, irrespective of ethnicity, area of residence, social status and on specified therapy protocols as mentioned in study design were included in the study.

#### Exclusion criteria

All hypertensive women having mental health issues affecting cognition, more than two co-morbid conditions and not willing to participate in the study were excluded from the study. No exclusion was made based on patient’s altered lab values.

### Variables

#### Treatments

Out of 300 hypertensive women, 40 were on Losartan potassium (LTN) 50 mg daily, 107 were on LTN + hydrochlorothiazide (HCT) 50/12.5 mg daily, 95 were on Nifedipine GITS 30 or 60 mg daily and 58 were on Nifedipine GITS + LTN + HCT 30 or 60 mg + 50/12.5 mg daily, all for the period of 12 months (Fig. 1).

#### Outcome measures

The primary efficacy endpoints were mean changes in systolic (SBP) and diastolic blood pressure (DBP) from the baseline values in each arm measured at each follow up till final follow up. BP was measured using manual BP measuring devices.

#### Safety endpoints

Safety parameters were estimated based on the reported frequencies of treatment related adverse reactions (ADRs). All adverse effects that occurred throughout the study period were recorded and evaluated for their seriousness and relation to the drugs. Notable ADRs, documented based on their associations with treatment protocols, include, dry cough, headache, orthostatic hypotension and edema.

#### Life style measures

##### Physical activity

A daily physical activity of 30 min was considered normal, while a daily activity of less than 30 min was considered as low.

##### Food consumption

Food consumption was documented to estimate their possible association with hypertension under the following sub-headings.;

- *Red or white meat:* consumption of red or white meat was scored as 1–2 times and > 2 times in a day – at least 35–70 g was considered 1–2 times per day, while more than 70 g was considered > 2 times a day.
- *Vegetables and fruits:* it was recorded as 1–2 times and 2–3 times a day – at least 1/3rd portion of the meal or 1–1.5 cup of vegetables & fruits, fresh or cooked was considered as 1–2 time a day, while more than twice was considered > 2 times a day.
- *Salt Intake:* Salt intake was documented as normal if daily intake was equal to 1 teaspoon. i-e., 6 g, while salt intake of ≤4.5 g was considered low.
- *Fat Intake:* consumption of trans and saturated food, snacks, fast food, creamers and cakes, was considered high fat consumption, while avoiding these food items was documented taken as low fat consumption [22]..

### Data collection

Data collection form was designed fulfilling all the necessary objectives of the study. Utilizing patient’s medical files, patient’s baseline demographics (name, age, gender, weight, address, BMI, occupation, marital status, and number of children), lifestyle patterns (physical activity and food consumption), clinical variables (systolic blood pressure (SBP) and diastolic blood pressure (DBP) in mmHg, history of cardiovascular events, the presence of comorbidities and laboratory biochemical values (blood sedimentation rate (BSR), urea and creatinine) were documented. Moreover, history of illness, disease symptoms and possible drug related side effects were also recorded.

On every follow up, after every 3 months, again pertinent clinical, SBP and DBP, and laboratory biochemical data, i-e,. BSR, urea, creatinine and safety endpoints were documented under the supervision of a medical practitioner.

### Data analysis

The socio-demographic characteristics of the patients segregated to four different anti-hypertensive therapies were analyzed and compared using StataSE14 and SPSS (IBM, version 21). Descriptive statistics were performed to estimate the frequencies of all socio-demographic variables and food consumption using cross-tabulation. A linear mixed effect model was used to evaluate the changes over time in SBP, DBP, urea, serum creatinine, Hb and random blood glucose levels.

Data for the primary outcomes, collected at 5 different study time points, from baseline to final follow up, were assumed to be clustered within patients. It is therefore unreasonable to assume that these data were independent. To account for the clustering effect of these data, we fitted linear multilevel models. Thus, a two-level model with random intercept and random effect of time on patients at level 2 was considered. The models were used to assess the mean changes of the primary outcomes at each study time point relative to baseline. To understand the factors that were associated with being in the low or high risk group, binary logistic regression models within the generalized linear regression model (GLM) were fitted to the data. All models were fitted using StataSE 14. An alpha value of o.0.5 or less was considered statistically significant.

## Results

### Patient’s demographics

Patient’s basic characteristics are summarized in Table 1. Data suggested that frequency distribution was significantly different among all the treatment protocols with regards to age, mostly < 60 years of age (LTN; 85%, N-GITS; 49.5%, LTN + HCT; 71%, N-GITS+LTN + HCT; 51.7%, *p* <  0.01), family history; mostly had no familial link (LTN; 60%, N-GITS; 76.8%, LTN + HCT; 57%, N-GITS+LTN + HCT; 56.9%, *p* = 0.01), education; mostly having secondary education (*p* <  0.01) and physical activity (*p* <  0.01); more than 44% of the subjects in each treatment arm claimed to have normal physical activity (Table 1). Similarly, majority of the patients on protocols other than LTN were consuming red or white meat 1–2 times a day (N-GITS; 58.9%, LTN + HCT; 80.4%, N-GITS+LTN + HCT; 91.4%, *p* <  0.01) and had low salt intake (N-GITS; 53.7%, LTN + HCT; 73.8%, N-GITS+LTN + HCT; 86.2%, *p* <  0.01) (Table 1).

**Table 1:** Patient’s Basic Demographics and Life Style Patterns

Characteristics	Anti-hypertensive Protocols				***p***-values
	LTN *n* = 40 (%)	N-GITS *n* = 95 (%)	LTN + HCT *n* = 107 (%)	N-GITS + LTN + HCT *n* = 58 (%)	
**Age** (years)					
< 60	34 (85.0)	47 (49.5)	76 (71.0)	30 (51.7)	< 0.01**
≥ 60	6 (15.0)	48 (50.5)	31 (29.0)	28 (48.3)	
**Body Weight** (kg)					
≤ 65	19 (47.5)	40 (42.1)	58 (54.2)	22 (37.9)	0.17
> 65	21 (52.5)	55 (57.9)	49 (45.8)	36 (62.1)	
**Marital Status**					
Married	39 (97.5)	89 (93.7)	100 (93.5)	54 (93.1)	0.80
Single	1 (2.5)	6 (6.3)	7 (6.5)	4 (6.9)	
**Area of Residence**					
Urban	27 (67.5)	60 (63.2)	66 (61.7)	37 (63.8)	0.93
Rural	13 (32.5)	35 (36.8)	41 (38.3)	21 (36.2)	
**Number of Children**					
< 3	19 (47.5)	23 (24.2)	34 (31.8)	19 (32.8)	0.07
≥ 3	21 (52.5)	72 (75.8)	73 (68.2)	39 (67.2)	
**Family History**					
Yes	16 (40.0)	22 (23.2)	46 (43.0)	25 (43.1)	0.01*
No	24 (60.0)	73 (76.8)	61 (57.0)	33 (56.9)	
**Occupation**					
Housewife	32 (80.0)	93 (97.9)	99 (92.5)	56 (96.6)	< 0.01**
Employed	8 (20.0)	2 (2.1)	8 (7.5)	2 (3.4)	
**Education**					
Secondary	13 (32.5)	71 (74.7)	66 (61.7)	40 (69.0)	< 0.01**
Above Secondary	27 (67.5)	24 (25.3)	41 (38.3)	18 (31.0)	
**Food Consumption**					
*Red or White meat*					
1–2 times	17 (42.5)	56 (58.9)	86 (80.4)	53 (91.4)	< 0.01**
> 2 times	23 (57.5)	39 (41.1)	21 (19.6)	5 (8.6)	
*Vegetables and Fruits*					
1–2 times	8 (20.0)	12 (12.6)	9 (8.4)	6 (10.3)	0.26
> 2 times	32 (80.0)	83 (87.4)	98 (91.6)	52 (89.7)	
*Fat Intake*					
Low	25 (62.5)	76 (80.0)	90 (84.1)	56 (96.6)	< 0.01**
Normal	15 (37.5)	19 (20.0)	17 (15.9	2 (3.4)	
*Salt Intake*					
Low	12 (30.0)	51 (53.7)	79 (73.8)	50 (86.2)	< 0.01**
Normal	28 (70.0)	44 (46.3)	28 (26.2)	8 (13.8)	
**Physical Activity**					
Low	9 (22.5)	49 (51.6)	35 (32.7)	32 (55.2)	< 0.01**
Normal	31 (77.5)	46 (48.4)	72 (67.3)	26 (44.8)	

*Abbreviations*: *LTN* losartan potassium, *N-GITS* Nifedipine GITS, *HCT* hydrochlorothiazide

p-values: * ≤ 0.05, ** <  0.01

### Treatment outcomes and laboratory biochemical values; baseline vs follow ups

Data on treatment outcomes and laboratory biochemical values, baseline (BL) vs follow up, are summarized in Table S1. Data revealed that mean DBP and hemoglobin baseline values were not different among all four cohorts. However, considerable differences existed among cohorts in the mean baseline values of SBP (LTN; 141.87, N-GITS; 156.52, LTN + HCT; 153.73, LTN + N-GITS+HCT; 184.05 mmHg), serum creatinine, serum urea and blood glucose levels (Table S1). When it comes to treatment effects, in last (12 months) follow up, all four therapeutic regimens had significant impact on SBP (LTN: BL; 141.87, 12-months; 123.25, *p* = 0.001, N-GITS: BL; 156.52, 12-months; 127.63, *p* = 0.001, LTN + HCT: BL; 153.73, 12-months; 128.54, *p* = 0.001, LTN + N-GITS + HCT: BL; 184.05, 12-months; 133.88, *p* = 0.001) and DBP (LTN: BL; 90.12, 12- months; 80.83, *p* = 0.001, N-GITS: BL; 93.51, 12-months; 81.28, *p* = 0.001, LTN + HCT: BL; 92.22, 12-months; 82.12, *p* = 0.001, LTN + N-GITS + HCT: BL; 100.44, 12-months; 84.04, *p* = 0.001). As for laboratory biochemical values, compared to baseline vs 12-months, mean serum creatinine levels were increased in LTN group only and mean serum urea levels were increased in N-GITS (31.27, *p* = 0.01), LTN + HCT (30.85, *p* = 0.001) and LTN + N-GITS+HCT (36.03, *p* = 0.001) groups. No significant effects of all four regimens were observed on Hb levels (Table S1).

### Frequency of co-morbid conditions and therapy related side effects

As shown in Figure S1, diabetes was the most frequent single co-morbid condition (13.6%) followed by anemia (11%), angina (7.7%) and ischemic heart disease (3%). In more than one co-morbid category, diabetes + angina (2.7%) and diabetes + anemia (1.3%) were the notable co-morbid conditions (Fig. S1). As for the side effects, in combination therapy; N-GITS+ LTN + HCT, orthostatic hypertension (15.8%) was most frequently observed side effect followed by edema (6.9%) (Fig. S2). In LTN + HCT group, orthostatic hypertension (8.4%) and headache (8.4%) were reported with similar frequencies. While in mono-therapy, patients on N-GITS experienced edema (5.2%), headache (4.7%) and orthostatic hypertension (3.2%), yet with lower frequencies compared to combination therapy (Fig. S2).

### Association of lifestyle patterns with overall hypertensive and high risk population

As shown in Table 2, hypertension was significantly associated with red/white meat intake > 2 times a day (OR; 2, *p* = 0.01), weight ≥ 65 Kg (OR; 1.63, *p* = 0.04), vegetables and fruits intake > 2 times a day (OR; 3.34, *p* = 0.001), salt intake (OR; 2.48, *p* <  0.01) and physical activity (OR; 2.79, *p* = 0.001). When adjusted for covariates, only salt intake, vegetables and fruit intake, and physical activity demonstrated significant associations with hypertension (Table 2).

**Table 2:** Association of Lifestyle Patterns in Women with Hypertension

Factors	Bivariate analysis		Adjusted	
	OR (95% CI)	***p***-value	OR (95% CI)	***p***-value
**Salt intake/day** (*ref: Low*)				
Normal	2.48 (1.50, 4.11)	< 0.01**	2.27 (1.20, 4.31)	0.01*
**Red/White meat intake/day** (*ref:* 1*–2 times*)				
> 2 times	2 (1.19, 3.42)	0.01*	1.81 (1.1, 3.81)	0.57
**Vegetables and fruits intake/day** (*ref:* 1*–2 times*)				
> 2 times	3.34 (1.61, 7.3)	< 0.01**	3.27 (1.6, 6.71)	< 0.01**
**Fat intake/day** (*ref: Low*)				
Normal	1.54 (0.82, 2.86)	0.18	1.43 (0.77, 2.91)	0.75
**Weight** (kg) (*ref: < 65 kg*)				
≥ 65 kg	1.63 (1.03, 2.60)	0.04*	1.56 (0.93, 2.62)	0.10
**Physical activity** (*ref: Low*)				
Normal	2.79 (1.74, 4.50)	< 0.01**	2.16 (1.29, 3.62)	< 0.01**

p-values: * ≤ 0.05, ** <  0.01

**Fig. 2:**
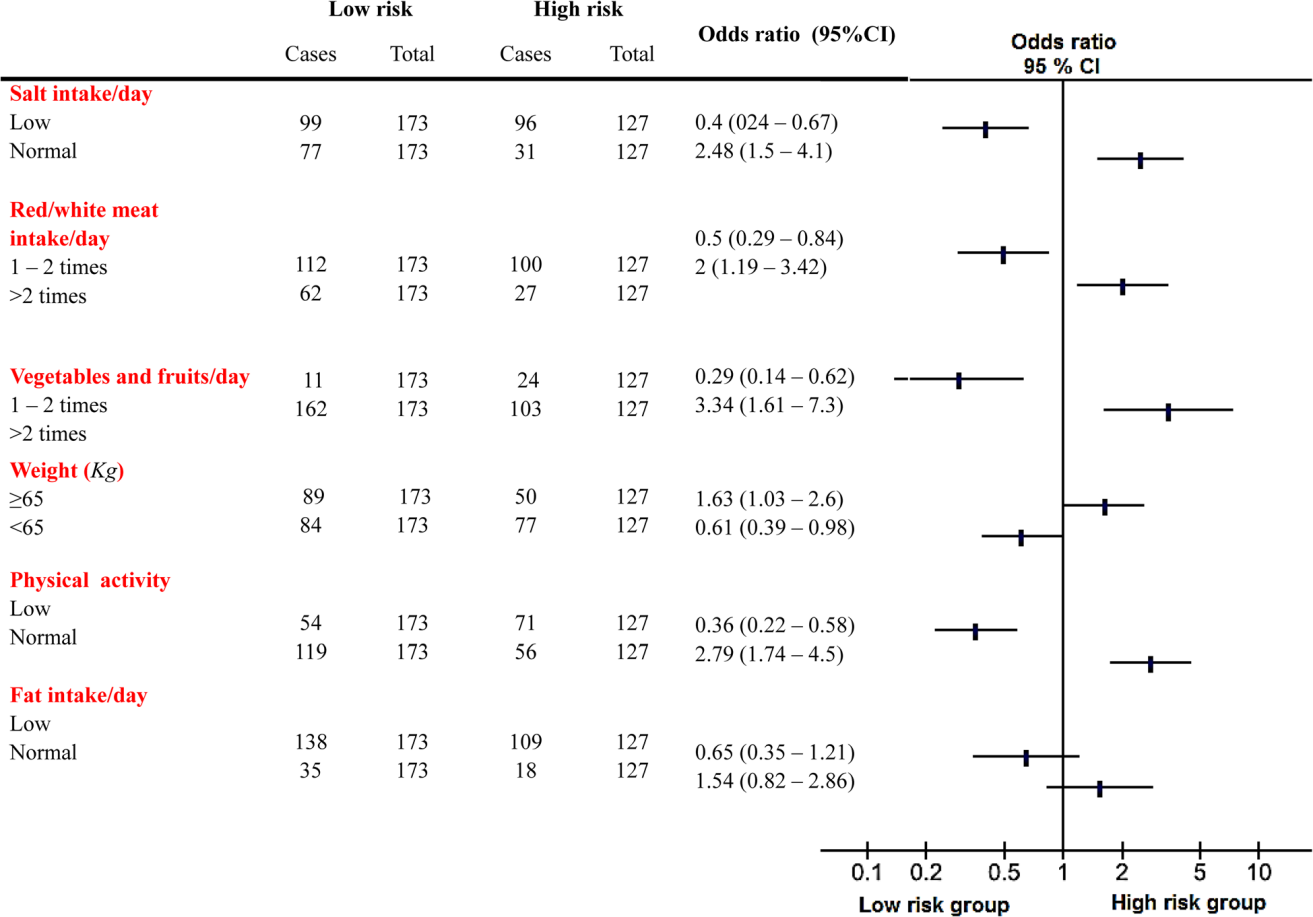
Forest Plot Showing Odd Ratios of Factors Associated with Hypertension in Women with Co-morbidities

When patients were segregated into low and high risk groups, as described in method section, forest plot revealed that high risk women were more likely to have normal salt intake (OR; 2.48, *ref;* low intake), meat intake > 2 times (OR; 2.02, *ref;* 1–2 times), vegetables and fruits intake > 2 times (OR; 3.43, *ref;* 1–2 times) and body weight ≥ 65Kg (OR;1.54, *ref;* <65Kg) compared to low risk subjects, despite normal physical activity and fat intake (OR; 1.54, *ref;* low intake) (Fig. 2).

### Changes in outcome measures at each follow up in hypertensive women

To examine the changes in outcome measures at each follow up in comparison to baseline values, we fitted models with second-order interaction between therapy and time in order to investigate the changes of the primary outcomes over time (Table 3). The mean changes reported in Table 3 are within various anti-hypertensive therapies relative to baseline, adjusted for socio-demographic factors. All treatment protocols, LTN, N-GITS, LTN + HCT and N-GITS + LTN + HCT demonstrated significant reduction in SBP starting at 3 months of follow up till 12 months, though maximum reduction was observed in N-GITS + LTN + HCT therapy group (β; − 50.17, *p* <  0.001) followed by N-GITS (β; − 28.89, *p* <  0.001), a similar trend was observed for DBP - N-GITS + LTN + HCT therapy group (β; − 16.55, *p* <  0.001) followed by N-GITS (β; − 12.21, *p* <  0.001) (Table 3). Compared to baseline laboratory biochemical values, at final follow up, 12 months, BSR levels exhibited maximum reduction in N-GITS (β; − 25.29, *p* <  0.001) and N-GITS + LTN + HCT therapy (β; − 24.93, *p* <  0.001) groups, while urea levels were increased in N-GITS + LTN + HCT (β; 3.47, *p* <  0.001) and LTN + HCT (β; 5.16, *p* <  0.001) groups (Table 3). Interestingly, only N-GITS treatment resulted in modest changes in blood urea and creatinine levels, which started to appear in 3rd follow up (Table 3).

**Table 3:** Therapy Related Changes in Outcome Measures at Each Follow up Compared to Baseline Using Linear Multilevel Regression Model

Changes in Outcome Measures	3 Months	6 Months			9 Months		12 Months	
	β (95% CI)	***p***-value	β (95% CI)	***p***-value	β (95% CI)	***p***-value	β (95% CI)	***p***-value
**Changes in SBP from baseline**								
LTN	−17.88 (−20.25, − 15.50)	< 0.01**	− 17.88 (−20.25, − 15.50)	< 0.01**	−16.38 (−18.75, −14.00)	< 0.01**	−18.63 (− 21.00, − 16.25)	< 0.01**
LTN + HCT	−25.79 (−27.25,-24.34)	< 0.01**	− 25.14 (−26.59, −23.69)	< 0.01**	−25.42 (−26.87, − 23.97)	< 0.01**	−26.45 (− 27.90, − 24.99)	< 0.01**
N-GITS	−28.53 (−30.07, − 26.989	< 0.01**	−28.16 (−29.70, − 26.61)	< 0.01**	−29.58 (−31.12, − 28.04)	< 0.01**	−28.89 (− 30.44, − 27.35)	< 0.01**
N-GITS + LTN + HCT	− 45.95 (− 47.92, − 43.97)	< 0.01**	− 48.88 (− 50.85, − 46.9)	< 0.01**	−49.74 (− 51.72, − 47.77)	< 0.01**	−50.17 (− 52.15, − 48.20)	< 0.01**
**Changes in DBP from baseline**								
LTN	−9.25 (− 11.18, − 7.32)	< 0.01**	−10.00 (− 11.93, −8.07)	< 0.01**	− 9.25 (− 11.18, − 7.32)	< 0.01**	− 9.75 (− 11.68, − 7.82)	< 0.01**
LTN + HCT	− 10.51 (− 11.69, − 9.34)	< 0.01**	−10.33 (− 11.50, − 9.15)	< 0.01**	−10.00 (− 11.18, − 8.82)	< 0.01**	−10.89 (− 12.07, − 9.71)	< 0.01**
N-GITS	− 12.32 (− 13.57, − 11.07)	< 0.01**	−11.74 (− 12.99, − 10.49)	< 0.01**	−11.58 (− 12.83, − 10.33)	< 0.01**	−12.21 (− 13.46, − 10.96)	< 0.01**
N-GITS + LTN + HCT	− 16.81 (− 18.41–15.21)	< 0.01**	−16.64 (− 18.24, − 15.04)	< 0.01**	−16.90 (− 18.50, − 15.30)	< 0.01**	−16.55 (− 18.15, − 14.95)	< 0.01**
**Changes in BSR from baseline**								
LTN	−9.63 (− 19.87, 0.62)	0.07	−5.33 (− 15.57, 4.92)	0.31	− 11.10 (− 21.35, − 0.85)	0.03*	− 13.23 (− 23.47, − 2.98)	0.01*
LTN + HCT	−4.96 (− 11.23, 1.30)	0.12	−7.21 (− 13.47, 0.94)	0.02*	− 10.73 (− 17.00, − 4.46)	< 0.01**	− 13.60 (− 19.86, − 7.33)	< 0.01**
N-GITS	−11.52 (− 18.17, −4.87)	< 0.01**	− 17.08 (− 23.73, − 10.43)	< 0.01**	− 21.39 (− 28.04, − 14.74)	< 0.01**	−25.29 (− 31.95, − 18.64)	< 0.01**
N-GITS + LTN + HCT	−13.38 (− 21.89, −4.87)	< 0.01**	− 17.62 (− 26.13, − 9.11)	< 0.01**	− 14.10 (− 22.62, − 5.59)	< 0.01**	−24.93 (− 33.44, − 16.42)	< 0.01**
**Changes in Urea from baseline**								
LTN	1.00 (− 0.66, 2.66)	0.24	2.35 (0.69, 4.01)	0.01*	2.03 (0.37, 3.68)	0.02*	2.65 (0.99, 4.31)	< 0.01**
LTN + HCT	0.38 (−0.64, 1.39)	0.47	1.29 (0.28, 2.30)	0.01*	2.19 (1.17, 3.20)	< 0.01**	3.47 (2.45, 4.48)	< 0.01**
N-GITS	−0.76 (− 1.83, 0.32)	0.17	0.71 (−0.37, 1.78)	0.20	1.39 (0.31, 2.47)	0.01*	1.81 (0.73, 2.89)	< 0.01**
N-GITS + LTN + HCT	0.72 (−0.65, 2.10)	0.30	2.93 (1.55, 4.31)	< 0.01**	3.60 (2.23, 4.98)	< 0.01**	5.16 (3.78, 6.53)	< 0.01**
**Changes in Creatinine from baseline**								
LTN	0.02 (−0.03, 0.06)	0.45	0.03 (−0.02, 0.07)	0.28	0.05 (−0.001, 0.09)	0.06	0.07 (0.02, 0.11)	< 0.01**
LTN + HCT	0.01 (−0.02, 0.03)	0.74	0.02 (−0.01, 0.05)	0.11	0.02 (−0.01, 0.05)	0.11	0.04 (0.01, 0.07)	< 0.01**
N-GITS	−0.03 (− 0.06, 0.003)	0.08	− 0.02 (− 0.05, 0.01)	0.11	−0.04 (− 0.07, − 0.01)	0.01*	−0.04 (− 0.07, − 0.01)	0.01*
N-GITS + LTN + HCT	−0.01 (− 0.05, 0.02)	0.47	0.01 (− 0.03, 0.04)	0.72	0.04 (0.002, 0.08)	0.04*	0.07 (0.03, 0.10)	< 0.01**

*Abbreviations*: *LTN* losartan potassium, *N-GITS* Nifedipine GITS, *HCT* hydrochlorothiazide, *SBP* systolic blood pressure, *DBP* diastolic blood pressure, *BSR* blood sedimentation rate

p-values: * ≤ 0.05, ** <  0.01

### Changes in outcome measures, low vs high risk hypertensive women

Table 4 showed the changes in outcome measures, i-e., SBP, DBP, urea, BSR and creatinine within and between low (LR) and high risk (HR) hypertensive women, adjusted for treatment effects, at each follow up. In low and high risk groups, compared to baseline, we observed significant reduction in SBP (*at 12 months:* LR; β; − 17.92, *p* <  0.001, HR; − 19.48, *p* <  0.001) and DBP (*at 12 months:* LR; β; − 9.49, *p* <  0.001, HR; − 10.12, *p* <  0.001) at each follow up, which became maximum at final follow up (12 months). In both low and high risk groups, compared to baseline, changes in urea and creatinine were observed after 2nd and 3rd follow ups (Table 4). But regarding changes between the groups, low vs high risk, only SBP (β; − 1.55, *p* = 0.03) demonstrated a significant change at final follow up (Table 4).

**Table 4:** Mean Changes in Outcomes Measures; Low vs High Risk Women, Adjusted for Treatment Effects

Changes in Outcome Measures from Baseline; Low vs. High Risk	3 Months		6 Months		9 Months		12 Months	
	β (95% CI)	***p***-value	β (95% CI)	***p***-value	β (95% CI)	***p***-value	β (95% CI)	***p***-value
**Changes in SBP relative to baseline**								
Low risk	−17.87 (− 20.53, − 15.23)	< 0.01**	−17.87 (− 20.51, − 15.24)	< 0.01**	−16.53 (− 19.17, − 13.88)	< 0.01**	− 17.92 (− 20.56, − 15.28)	< 0.01**
High risk	−17.87 (− 20.34, − 15.40)	< 0.01**	−17.87 (− 20.38, − 15.36)	< 0.01**	−16.19 (− 18.69, − 13.68)	< 0.01**	−19.48 (− 21.99, − 16.97)	< 0.01**
**Change between** (*ref: Low risk*)								
High risk	0.006 (− 1.32, 1.33)	0.98	0.009 (− 1.29, 1.30)	0.99	0.34 (− 1.01, 1.69)	0.62	− 1.55 (− 2.92, − 0.18)	0.03*
**Changes in DBP relative to baseline**								
Low risk	−8.49 (− 10.63, − 6.35)	< 0.01**	− 10.17 (− 12.28, − 8.05)	< 0.01**	− 9.25 (− 11.38, − 7.13)	< 0.01**	− 9.49 (− 11.62, − 7.37)	< 0.01**
High risk	−9.97 (− 11.97, − 7.98)	< 0.01**	−9.84 (− 11.87, − 7.82)	< 0.01**	−9.31 (− 11.33, − 7.28)	< 0.01**	− 10.12 (− 12.15, − 8.10)	< 0.01**
**Change between** (*ref: Low risk*)								
High risk	−1.57 (− 2.60, − 0.54)	< 0.01**	0.24 (− 0.78, 1.25)	0.65	− 0.14 (− 1.20, 0.92)	0.79	− 0.72 (− 1.79, 0.35)	0.19
**Changes in BSR relative to baseline**								
Low risk	−7.13 (− 18.65, 4.40)	0.23	−1.81 (− 13.31, 9.68)	0.76	− 8.75 (− 20.25, 2.76)	0.14	− 11.17 (− 22.67, 0.34)	0.06
High risk	−10.16 (−20.86, 0.53)	0.06	− 7.00 (− 17.91, 3.90)	0.21	− 11.30 (− 22.17, − 0.43)	0.04*	−13.06 (− 23.95, −2.17)	0.02*
**Change between** (*ref: Low risk*)								
High risk	0.98 (− 4.99, 6.95)	0.75	−1.17 (− 7.07, 4.73)	0.70	1.46 (− 4.67, 7.59)	0.64	2.12 (− 4.09, 8.34)	0.50
**Changes in Urea relative to baseline**								
Low risk	1.06 (−0.81, 2.93)	0.27	2.49 (0.62, 4.35)	0.01*	2.04 (0.18, 3.91)	0.03*	2.83 (0.97, 4.70)	< 0.01**
High risk	1.00 (−0.73, 2.73)	0.26	2.27 (0.50, 4.03)	0.01*	2.09 (0.33, 3.85)	0.02*	2.51 (0.75, 4.28)	0.01*
**Change between** (*ref: Low risk*)								
High risk	0.07 (−0.90, 1.04)	0.89	−0.09 (−1.05, 0.87)	0.86	0.18 (−0.82, 1.17)	0.73	−0.19 (− 1.20, 0.82)	0.71
**Changes in Creatinine relative to baseline**								
Low risk	0.01 (−0.04, 0.06)	0.77	0.01 (−0.04, 0.07)	0.58	0.05 (0.004, 0.11)	0.04*	0.07 (0.02, 0.12)	0.01*
High risk	0.01 (−0.02, 0.03)	0.57	0.03 (−0.01, 0.08)	0.17	0.03 (−0.02, 0.08)	0.26	0.06 (0.01, 0.11)	0.01*
**Change between** (*ref: Low risk*)								
High risk	0.008 (−0.02, 0.03)	0.57	0.01 (−0.01, 0.04)	0.40	−0.03 (− 0.06, − 0.01)	0.01*	−0.01 (− 0.04, 0.01)	0.36

*Abbreviations*: *LTN* losartan potassium, *N-GITS* Nifedipine GITS, *HCT* hydrochlorothiazide, *SBP* systolic blood pressure, *DBP* diastolic blood pressure, *BSR* blood sedimentation rate

*p*-values * ≤ 0.05, ** < 0.01

## Discussion

Hypertension is a major risk factor of cardiovascular diseases (CVDs) and a major contributor to CVDs related deaths, approximately one death per minute among women in the United States [23]. According to recent estimates from Punjab, Pakistan, the prevalence of hypertension in women is 41%, almost 10% higher than men [20]. However, not a single study from Pakistan has been reported to estimate the clinical efficacy of different anti-hypertensive agents in women of Punjab, Pakistan. In the present study it was observed that among hypertensive women of Punjab, Pakistan, diabetes is the most common co-morbid condition followed by anemia, angina and ischemic heart disease. Life style and dietary patterns demonstrated significant associations with high risk hypertensive subjects, such as consumption of salt, fat, red/white meat, vegetables and fruits, and physical activity in comparison to low risk hypertensive subjects. Among the four treatment cohorts, combination regimen; N-GITS + LTN + HCT, and single agent N-GITS demonstrated improved anti-hypertensive effects, on both SBP and DBP, however, the treatment related side effects, orthostatic hypotension and edema, were less frequently observed with monotherapy compared to combination therapy. Only N-GITS exhibited minimal effects on serum urea and creatinine levels.

As reported previously, with advance aging, in post-menopausal women above 65 years of age, the percentage of women with hypertension is higher compared to men [23]. We also observed that 60% hypertensive women were between 50 and 64 years of age compared to only 20.3% hypertensive women under the age of 50 years. Several previous researches have been conducted to assess dietary approaches in the management of hypertension and to estimate an association between diet and hypertension [24], yet the association cannot be ascribed to a single food item or nutrient which makes it a more composite risk factor in South Asians due to considerable variations in diet within and between South Asian population [25]. In this context, salt consumption in South Asians are generally higher that not only effects blood pressure but also increases the risk of stroke and cardiovascular diseases by altering arterial stiffness – contributing towards resistant hypertension in patients that are considered salt sensitive [26]. We found that compared to low salt consumption, patients consuming normal salt were considered high risk population corroborating findings of a systematic review that consuming lower salt can reduce blood pressure and subsequent risk of cardiovascular disease [27]. Fruits and vegetables intake have been shown to reduce blood pressure in number of studies [28], however, a study from Pakistan showed no association between fruit and vegetables consumption and lower risk of hypertension [24]. We found that compared to low intake, higher consumption of vegetables and fruits, red/white meat and high fat diet could frame women as high risk population. This could possibly be ascribed to the cooking methods used by South Asians, i-e., stir frying, overcooking of vegetables, use of animal saturated fats/ desi ghee (extracted from butter) in daily vegetables cooking and poor ascertainment regarding consumption of fruits and vegetables, whether cooked or uncooked. Our data regarding meat consumption and risk of hypertension is in complete agreement with previously reported data that higher consumption of meat, particularly red, is strongly associated with higher risk of cardiovascular diseases in women [29].

According to JNC 8 guidelines, first line antihypertensive therapy should consist of thiazide-type diuretic, calcium channel blockers, angiotensin-converting enzyme inhibitor (ACEIs), or an angiotensin receptor blocker (ARB) [30]. The European society of hypertension and European society of cardiology guidelines recommend the use of combination therapy in majority of patients – with calcium channel blockers being the most preferred combination [11]. Likewise, randomized and observational studies have shown that CCB, Nifedipine-GITS, is effective both as monotherapy and in combination [31, 32]. In this context, the TALENT study demonstrated that the use of Nifedipine GITS 20 mg in combination with telmisartan 80 mg provided better and earlier blood pressure control compared to monotherapy [31]. Similarly, AdADOSE, a 12 week multicenter, prospective, observational study, suggested that a combination therapy with Nifedipine-GITS was more effective in reducing systolic (SBP) and diastolic blood pressure (DBP) compared to other therapeutic choices along with low frequency of treatment related adverse effects [17]. Similar to these findings, we found that Nifedipine-GITS, compared to baseline, either in combination or alone, demonstrated significant reductions in SBP and DBP starting from 1st follow till the last follow up, yet maximum reduction was observed when Nifedipine-GITS was used in combination with other drugs, i-e., losartan (LTN) and hydrochlorothiazide (HCT). Moreover, changes in blood sedimentation rate (BSR) with Nifedipine-GITS alone or in combination was almost similar. Compared to combination therapy, Nifedipine-GITS alone exhibited minimal changes in urea and creatinine levels as reported previously [33]. Additionally, Nefidipine-GITS alone exhibited minimal side effects, such as dry cough, headache, orthostatic hypotension and odema, however when used with fixed dose combination, losartan and hydrochlorothiazide, orthostatic hypotension was significantly higher followed by odema. Nonetheless, contrary to our findings, combination of Nifedipine-GITS and candesartan exhibited improved safety profiles with lower incidence of vasodilatory adverse effects, such as odema, and headache [34]. In another study, ADVISE, combination of Nifedipine GITS + Valsartan compared to Valsartan alone demonstrated better and consistent blood pressure control in Asian population [15], but with a few vasodilatory side effects, as observed in our study, mostly with combination therapy, i-e., N-GITS + LTN + HCT. Hence, the more sever vasodilatory adverse events in our study, odema and orthostatic hypotension, could be attributed to the use of fixed dose triple combination, i-e., losartan, hydrochlorothiazide in combination with Nifedipine GITS. Thus, data from our study and of others clearly demonstrated that Nifedipine GITS in combination with angiotensin II receptor blockers and hydrochlorothiazide rendered better blood pressure control in hypertensive patients, particularly women, but may increase the frequency of vasodialtory effects.

### Study limitations

Our study has a few limitations; observational design, subjects were observed over a period of time but were not allowed to intervene, and collection of data from a single tertiary care facility that may limit the generalization of study results. Additionally, no information was available regarding financial and other stressors affecting blood pressure control. Similarly, information on the use of traditional remedies, very common in Pakistan, and their contribution in the control of blood pressure cannot be ascertained. Moreover, not a single literature report from Pakistan on the studied topic was available to directly compare our results.

### Implications for practice/policy

Pakistan, a male dominant society, where females seldom enjoy full rights and access to opportunities with regards to very basic needs. The situation is even worse in health sector due to lack of female doctors and cultural forces limiting the access to health facilities. In Pakistan, hypertensive males and females, irrespective of risk level, are treated using routine but similar treatment algorithms and non-pharmacological approaches. Additionally, not a single primary or tertiary care facility utilizes gender specific anti-hypertensive protocols. Our findings, the first report from Pakistan, clearly demonstrated that in high risk hypertensive women, compared to losartan, Nifedipine-GITS would be more suitable single agent choice with minimal side effects. Moreover, combining Nifedipine-GITS with fixed dose combination, Losartan + Hydrochlorothiazide, would provide improved blood pressure control in sever and resistant/high risk hypertensive women. Thus, these findings would be of interest to clinicians in implementing lifestyle modifications and selection of antihypertensive therapy in hypertensive women of Pakistan, and would also impact anti-hypertensive drug enlistment and procurement criterion, particularly for hospitals with higher influx of hypertensive women.

## Conclusion

In conclusion, our data clearly demonstrated that nifedipine-GITS alone is more efficacious in lowering systolic and diastolic BP in high and low-risk female patients than losartan alone, with improved renal protection. Additionally, the combination of nifedipine GITS with fixed dose losartan + HCT had improved blood pressure lowering effects but with higher frequency of vasodilatory side effects, such as orthostatic hypotension and edema. Thus, it may be appropriate to initially manage high risk hypertensive women on a single agent, i-e., Nifedipine-GITS in comparison to Losartan, having better clinical efficacy and safety profiles, if counselled properly with regards to life style and dietary modifications.

## Supplementary information

**Additional file 1 Table S1.** Population clinical and laboratory biochemical values of four anti-hypertensive therapies; baseline vs follow ups. **Figure S1.** Macrograph Showing Frequency of Co-morbidities in Hypertensive Women of Lahore, Punjab, Pakistan. **Figure S2.** Macrograph Showing Frequency of Therapy Related Adverse Effects in Hypertensive Women.

## Data Availability

The data sets used in the study can be provided upon request to corresponding author.

## Abbreviations

Nifedipine-GITS: Nifedipine gastrointestinal system
HIS: Hospital information system
SBP: Systolic blood pressure
ACEIs: Angiotensin converting enzyme inhibitor
BP: Blood pressue
LTN: Losartan potassium
HCT: Hydrochlorothiazide
DBP: Diastolic blood pressure
ADR: Adverse drug reaction
GLM: Generalized linear regression model
SPSS: Statistical software for the social sciences
BSR: Blood sedimentation rate
CVDs: Cardiovascular diseases
CCB: Calcium channel blocker
ARB: Angiotensin receptor blockers
OR: Odds ratio
JNC6: Joint national committee 6
