# Supplementary material for: Clinical efficacy of various anti-hypertensive regimens in hypertensive women of Punjab; a longitudinal cohort study
Source: BMC Womens Health. 2020 Aug 1;20:161. doi: 10.1186/s12905-020-01033-2 (PMC7395419; doi:10.1186/s12905-020-01033-2)

**Table S1.** Population clinical and laboratory biochemical values of four anti-hypertensive therapies; baseline vs follow ups

| Clinical variables | Regimens | Baseline | Follow ups | | | |
| --- | --- | --- | --- | --- | --- | --- |
|  |  |  | **3 months** | **6 months** | **9 months** | **12 months** |
|  |  | *Mean values* | *Mean values* | *Mean Values* | *Mean values* | *Mean values* |
| Mean Systolic Blood Pressure (*mmHg*) | *Losartan* | 141.87 | 124  *(p=0.001)* | 124  *(p=0.001)* | 125  *(p=0.001)* | 123.25  *(p=0.001)* |
|  | *Nifedipine GITZ* | 156.52 | 128  *(p=0.001)* | 128.37  *(p=0.001)* | 126.95  *(p=0.001)* | 127.63  *(p=0.001)* |
|  | *Losartan+HCT* | 153.73 | 127.97  *(p=0.001)* | 128.58  *(p=0.001)* | 128.35  *(p=0.001)* | 128.54  *(p=0.001)* |
|  | *Nifedipine GITZ, Losartan+HCT* | 184.05 | 138.103  *(p=0.001)* | 135.17  *(p=0.001)* | 134.31  *(p=0.001)* | 133.88  *(p=0.001)* |
| Mean Diastolic blood pressure (*mmHg*) | *Losartan* | 90.12 | 80.88 *(p=0.001)* | 80.13 *(p=0.001)* | 80.875 *(p=0.001)* | 80.38 *(p=0.001)* |
|  | *Nifedipine GITZ* | 93.51 | 82.02  *(p=0.001)* | 81.70  *(p=0.001)* | 81.92  *(p=0.001)* | 81.28  *(p=0.001)* |
|  | *Losartan+HCT* | 92.22 | 81.75  *(p=0.001)* | 81.89  *(p=0.001)* | 82.26  *(p=0.001)* | 82.12  *(p=0.001)* |
|  | *Nifedipine GITZ, Losartan+HCT* | 100.44 | 85.26  *(p=0.001)* | 84.04  *(p=0.001)* | 83.69  *(p=0.001)* | 84.04  *(p=0.001)* |
| Mean serum creatinine levels (*mg/dL*) | *Losartan* | 0.68 | 0.69  *(p=1.0)* | 0.70  *(p=1.0)* | 0.72  *(p=0.43)* | 0.74  *(p=0.05)* |
|  | *Nifedipine GITZ* | 0.93 | 0.904  (p=0.51) | 0.91  (p=1.0) | 0.89  *(p=0.78)* | 0.89  *(p=0.99)* |
|  | *Losartan+HCT* | 0.90 | 0.91  *(p=1.0)* | 0.93  *(p=1.0)* | 0.93  *(p=1.0)* | 0.95  *(p=0.54)* |
|  | *Nifedipine GITZ, Losartan+HCT* | 1.01 | 0.99  *(p=1.0)* | 1.02  *(p=1.0)* | 1.05  *(p=0.51)* | 1.07  *(p=0.09)* |
| Mean serum urea levels (*mg/dL*) | *Losartan* | 26.40 | 27.40 *(p=1.0)* | 28.75 *(p=0.66)* | 28.43  *(p= 0.32)* | 29.05  *(p=0.27)* |
|  | *Nifedipine GITZ* | 29.46 | 28.71  *(p=1.0)* | 30.17  *(p=1.0)* | 30.85  (p=0.27) | 31.27  *(p=0.01)* |
|  | *Losartan+HCT* | 27.35 | 27.72  *(p=1.0)* | 28.64  *(p=0.21)* | 29.53  *(p=0.001)* | 30.81  *(p=0.001)* |
|  | *Nifedipine GITZ, Losartan+HCT* | 30.88 | 31.60  *(p=1.0)* | 33.81  *(p=0.001)* | 34.483  *(p=0.001)* | 36.03  *(p=0.001)* |
| Mean blood glucose random (*mg/dL*) | *Losartan* | 127.5 | 117.9 *(p=0.59)* | 122.2 *(p=1.0)* | 116.43 *(p=0.58)* | 114.3 *(p=0.29)* |
|  | *Nifedipine GITZ* | 154.16 | 142.64  *(p=0.01)* | 137.04  *(p=.004)* | 132.77  *(p=0.001)* | 128.86  *(p=0.001)* |
|  | *Losartan+HCT* | 130.39 | 125.43  *(p=0.65)* | 123.187  *(p=0.27)* | 119.66  *(p=0.03)* | 116.79  *(p=0.07)* |
|  | *Nifedipine GITZ, Losartan+HCT* | 148.86 | 135.48  *(p=0.02)* | 131.24  *(p=0.01)* | 134.76  *(p=0.31)* | 123.93  *(p=0.005)* |
| Mean hemoglobin (*mg/dL*) | *Losartan* | 12.71 | 12.71  *(p=0.65)* | 12.84  *(p=0.83)* | 12.85  *(p=0.07)* | 12.90  *(p=0.09)* |
|  | *Nifedipine GITZ* | 12.51 | 12.49  *(p=1.0)* | 12.56  *(p=1.0)* | 12.53  *(p=1.0)* | 12.59  *(p=0.93)* |
|  | *Losartan+HCT* | 12.69 | 12.62  *(p=.71)* | 12.704  *(p=1.0)* | 12.67  *(p=1.0)* | 12.76  *(p=1.0)* |
|  | *Nifedipine GITZ, Losartan+HCT* | 12.76 | 12.721  *(p=1.0)* | 12.89  *(p=1.0)* | 12.86  *(p=1.0)* | 12.87  *(p=1.0)* |

**Figure S1.** Macrograph Showing Frequency of Co-morbidities in Hypertensive Women of Lahore, Punjab, Pakistan


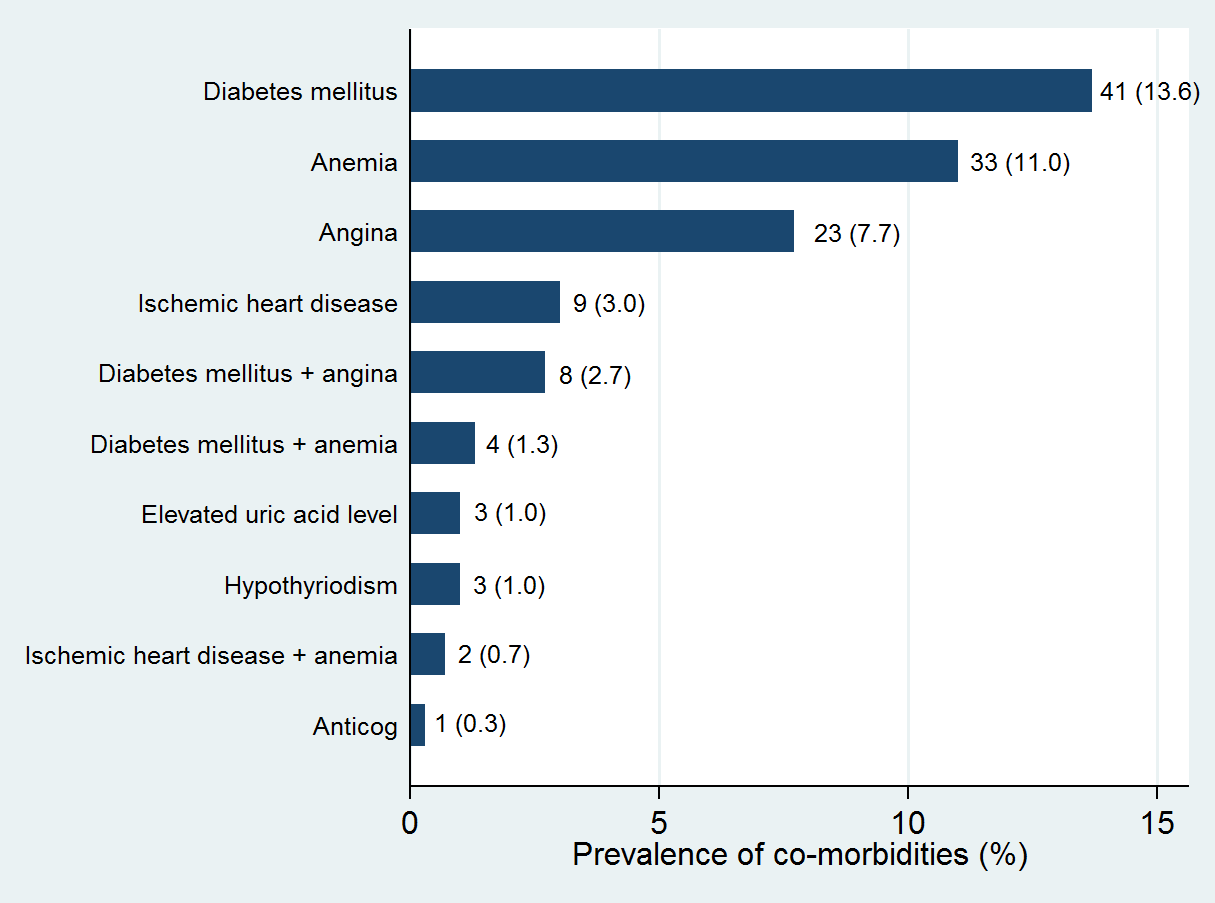


**Figure S2.** Macrograph Showing Frequency of Therapy Related Adverse Effects in Hypertensive Women.


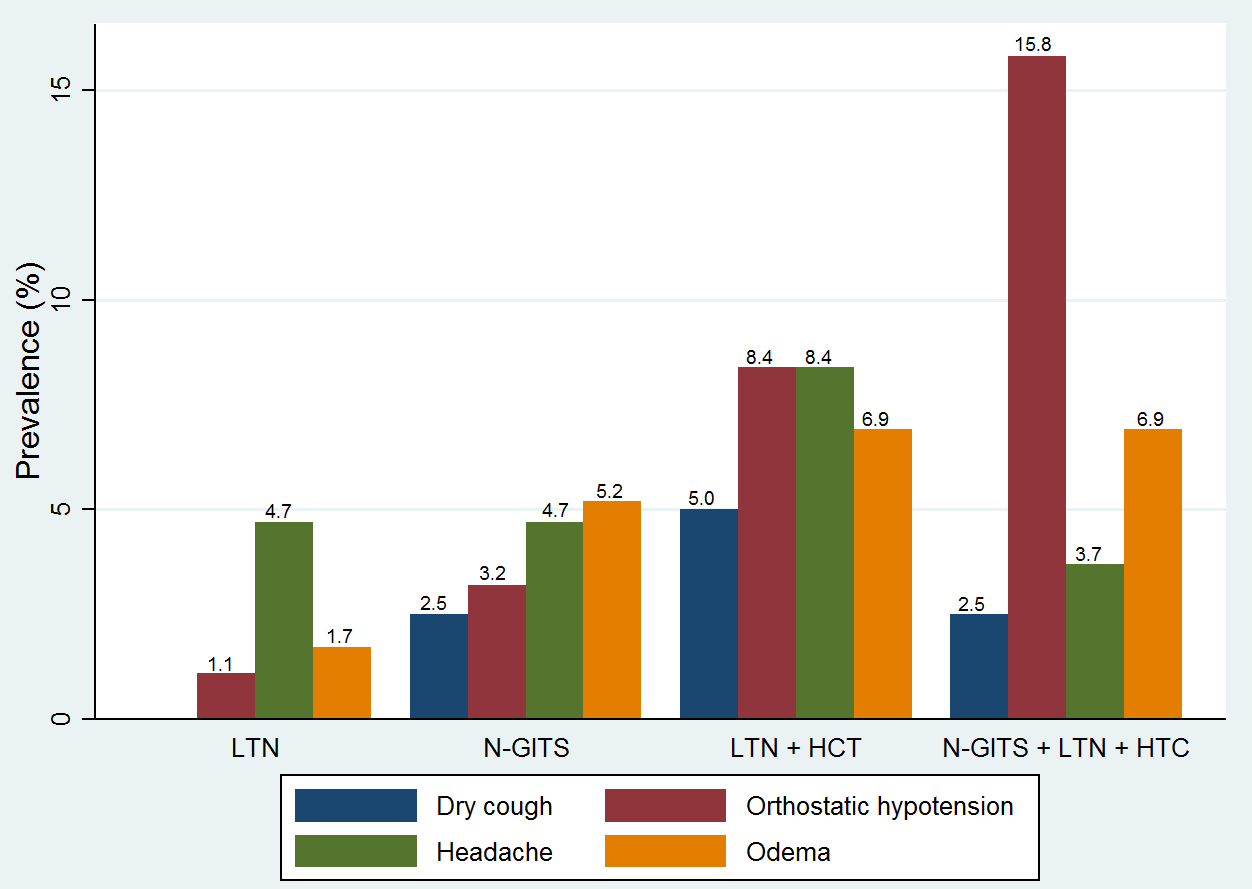

Supplement: Supplementary file 1 — Additional file 1 Table S1. Population clinical and laboratory biochemical values of four anti-hypertensive therapies; baseline vs follow ups. Figure S1. Macrograph Showing Frequency of Co-morbidities in Hypertensive Women of Lahore, Punjab, Pakistan. Figure S2. Macrograph Showing Frequency of Therapy Related Adverse Effects in Hypertensive Women. [file 12905_2020_1033_MOESM1_ESM.docx]
